# Rhizobia-legume symbiosis mediates direct and indirect interactions between plants, herbivores and their parasitoids

**DOI:** 10.1016/j.heliyon.2024.e27815

**Published:** 2024-03-13

**Authors:** Carlos Bustos-Segura, Adrienne L. Godschalx, Lucas Malacari, Fanny Deiss, Sergio Rasmann, Daniel J. Ballhorn, Betty Benrey

**Affiliations:** a Laboratory of Evolutionary Entomology, Institute of Biology, University of Neuchâtel, Neuchâtel, Switzerland; b Sorbonne Université, Institut National de Recherche pour L'Agriculture, L'Alimentation et L'Environnement, CNRS, Institut de Recherche pour le Développement, Université Paris-Est-Créteil-Val-de-Marne, Université Paris Cité, Institut D’Ecologie et des Sciences de L’Environnement de Paris, Versailles, 78026, France; c Laboratory of Functional Ecology, Institute of Biology, University of Neuchâtel, Neuchâtel, Switzerland; d Department of Biology, Portland State University, Portland, OR, USA

**Keywords:** Rhizobia, Phaseolus, Below-ground interactions, Indirect effects, Extrafloral nectar, Multitrophic interactions

## Abstract

Microorganisms associated with plant roots significantly impact the quality and quantity of plant defences. However, the bottom-up effects of soil microbes on the aboveground multitrophic interactions remain largely under studied. To address this gap, we investigated the chemically-mediated effects of nitrogen-fixing rhizobia on legume-herbivore-parasitoid multitrophic interactions. To address this, we initially examined the cascading effects of the rhizobia bean association on herbivore caterpillars, their parasitoids, and subsequently investigated how rhizobia influence on plant volatiles and extrafloral nectar. Our goal was to understand how these plant-mediated effects can affect parasitoids. Lima bean plants (*Phaseoulus lunatus*) inoculated with rhizobia exhibited better growth, and the number of root nodules positively correlated with defensive cyanogenic compounds. Despite increase of these chemical defences, *Spodoptera latifascia* caterpillars preferred to feed and grew faster on rhizobia-inoculated plants. Moreover, the emission of plant volatiles after leaf damage showed distinct patterns between inoculation treatments, with inoculated plants producing more sesquiterpenes and benzyl nitrile than non-inoculated plants. Despite these differences, *Euplectrus platyhypenae* parasitoid wasps were similarly attracted to rhizobia- or no rhizobia-treated plants. Yet, the oviposition and offspring development of *E. platyhypenae* was better on caterpillars fed with rhizobia-inoculated plants. We additionally show that rhizobia-inoculated common bean plants (*Phaseolus vulgaris*) produced more extrafloral nectar, with higher hydrocarbon concentration, than non-inoculated plants. Consequently, parasitoids performed better when fed with extrafloral nectar from rhizobia-inoculated plants. While the overall effects of bean-rhizobia symbiosis on caterpillars were positive, rhizobia also indirectly benefited parasitoids through the caterpillar host, and directly through the improved production of high quality extrafloral nectar. This study underscores the importance of exploring diverse facets and chemical mechanisms that influence the dynamics between herbivores and predators. This knowledge is crucial for gaining a comprehensive understanding of the ecological implications of rhizobia symbiosis on these interactions.

## 1 Introduction

Nitrogen-fixing bacteria, such as rhizobia, form a symbiotic relationship with plants, primarily legumes [1]. In this relationship, the plant provides shelter and carbon compounds from photosynthesis to the bacteria, while the bacteria, in turn, convert atmospheric nitrogen into plant-utilizable forms, such as ammonia [2], which allows legumes to grow in nitrogen-poor soils [3]. One of the major benefits arising from this symbiotic relationship, is the notable enhancement of plant growth and fitness [4]. This symbiosis also exerts a positive influence on plant nutrition and crop yields, ultimately contributing to a decreased reliance on synthetic nitrogen fertilizers [[5], [6], [7]]. This beneficial effect is particularly notable in sustainable agroecosystems that use intercropping practices [3,8].

However, these mutualisms can also lead to adverse effects on plants. More precisely, nitrogen-fixation, which increases nitrogen levels in plant tissues, benefits herbivores, as they typically suffer from nitrogen deficiency, both above and belowground [[9], [10], [11], [12]]. For example, soybean plants with high nodulation significantly improved the performance of *Helicoverpa zea* larvae [13], and rhizobia positively affected *Spodoptera littoralis* larvae when feeding on non-cyanogenic strains of white clover, *Trifolium repens.* [14] In turn, increased nutrient availability from nitrogen fixation can also lead to the production of higher levels of secondary metabolites, with potential adverse effects on herbivores [[14], [15], [16], [17], [18]]. Rhizobia-colonized lima bean plants, for example, exhibited increased production of cyanogenic glycoside (CNGs), leading to reduced damage from Mexican bean beetles (*Epilachna varivestis*) [15]. Additionally, beetles from the same species were repelled by volatile organic compounds (VOCs) emitted from rhizobia-colonized plants [16]. Hence, the effects of the rhizobia-plant symbiosis on herbivores are mixed, with some studies showing positive and others reporting negative effects.

This scenario can become more intricate when higher trophic levels are considered, as the symbiosis between beans and rhizobia has been shown to also impact the natural enemies of herbivores in various ways [19]. On one hand, the rhizobia-enhanced plant growth and survival, can increase the diversity and abundance of natural enemies in the ecosystem, resulting in increased herbivore mortality and ultimately leading to reduced herbivore populations [20]. On the other hand, the quality of the herbivores can affect parasitoid performance, which is influenced by altered primary and secondary metabolites in plants. For example, a nutritious host plant can produce large and healthy herbivores, thereby supporting the growth and development of parasitoid progeny [21]. Conversely, the rhizobia-mediated increase in the production of secondary metabolites, which can accumulate in the bodies of the herbivores, can indirectly impact the survival of natural enemies, including parasitoids and predators that prey on these herbivores [19,22].

In this context, parasitoids are particularly interesting as their performance is highly dependent on the quality of their herbivore host. However, our understanding of the effects of rhizobia-legume symbiosis on parasitoid behaviour and performance remains limited. Accordingly, the presence of rhizobia in the root system of legume plants can additionally modify the composition and emission of VOCs produced by plants [16,23]. This alteration may, in turn, influence the ability of parasitoids to locate their hosts, leading to changes in their foraging behaviour and overall effectiveness as biocontrol agents. But VOCs are not the only type of indirect defences. Some plant species, such as most legumes, can also attract natural enemies via the production of extrafloral nectar [24,25]. Extrafloral nectar plays a crucial role in providing carbohydrates to natural enemies of predators, serving as an indirect form of biological defence [26]. Therefore, the effects of rhizobia mediated on herbivores and their predators/parasitoids should be viewed as a continuum encompassing trait-mediated direct and indirect interactions. Nevertheless, this perspective remains insufficiently explored, and our understanding of the complete ecological consequences of plant-rhizobia symbiosis is still incomplete. Addressing the mechanisms driving such network of interactions can help foster both fundamental research in community ecology and applied research in biological control of insect pests.

In this study, we examined the effect of the bean-rhizobia symbiosis on plant-herbivore-parasitoid interactions. Firstly, using wild lima bean plants (*Phaseolus lunatus)*, we assessed plant growth, as well as the production of direct (cyanogenic glycosides) and indirect defences (VOCs) in plants with and without rhizobia. Then, we examined the effect of rhizobia on the preference and performance of *Spodoptera latifascia* caterpillars and their parasitoid, *Euplectrus platyhypenae*. Furthermore, with plants of the black common bean (*Phaseolus vulgaris)* we explored the direct interactions between these plants and the parasitoid *E. platyhypenae*. In this context, we determined the influence of rhizobia on the production and quality of extrafloral nectar and how it impacted the survival and performance of the parasitoid. With this series of experiments, we asked the following questions: 1) What is the impact of the rhizobia-bean symbiosis on plant growth and the production of direct (cyanogenic glycosides) and indirect defences (volatile compounds)? 2) How does the rhizobia-bean symbiosis affect the herbivores and parasitoids, as well as their host selection behaviour? 3) How does the bean-rhizobia symbiosis influence the quantity and quality of extrafloral nectar produced by plants, and what are the resulting consequences for the survival and performance of parasitoids? By exploring the impact of rhizobia on plant growth, direct defences, herbivore responses, and two different indirect plant defences, along with their consequences for parasitoid behaviour and survival, we aim to enhance our understanding of how soil beneficial microbes play a role in aboveground multitrophic interactions.

## 2 Materials and methods

### 2.1 Study species

#### *Plants*

The genus *Phaseolus* is native to the Americas, with a distribution ranging from Mexico and Central America to the Pacific coast of South America. Lima bean, *Phaseolus lunatus* and the black common bean, *Phaseolus vulgaris* are amongst the five species that have been domesticated and their symbiosis with rhizobia has been extensively studied [27]. Wild and cultivated plants of these two species have been found to be naturally nodulated by diverse species and strains of rhizobia [[27], [28], [29], [30]], overall inducing beneficial effects on plants, such as plant growth and yield enhancement [31].

The common bean (*P. vulgaris*) is an important crop species that produces bean seeds with a high protein content. Its centre of origin has been located in Mesoamerica and it is distributed naturally from Mexico to the south of Argentina [32]. The main rhizobia species associated with *P. vulgaris* is *Rhizobium etli.* [27] However, other species are known to nodulate in *P. vulgaris.* [33] This unspecific nodulation enables *P. vulgaris* to nodulate in diverse habitats, including non-native environments.

Lima bean, *P. lunatus*, has been used as a model system in studies on direct and indirect plant defences [25,34,35]. It is the only *Phaseolus* species known to contain cyanogenic glycosides (CNGs), acting as feeding deterrents or inhibitors against non-adapted insects [[36], [37], [38]]. Both *P. lunatus* and *P. vulgaris* have been shown to emit herbivore-induced VOCs that attract natural enemies [[39], [40], [41], [42]], and produce extrafloral nectar that serves as food for ants [17,[43], [44], [45]] and parasitic wasps [46]. *P. lunatus* nodulates with several strains of *Rhizobium* and *Bradyrhizobium* species [16]. However, these symbionts have a restricted distribution and are not found in European soils.

Wild lima bean (*P. lunatus*) plants were grown from seeds collected in December 2018 from a natural population present at the experimental campus of the Universidad del Mar, located 15 km northwest of the city of Puerto Escondido [47] (Oaxaca, Mexico, 15°55′27.1″N, 97°09′05.3″W), while *P. vulgaris* plants were grown from seeds obtained from Ferme de Sainte Marthe (variety Coco noir Staragorosky, France). Seeds of *P. lunatus* were scarified with sandpaper before sowing to increase germination. Seeds of both bean species were placed in Petri dishes lined with moist paper towel, in an incubator for 24 h (Conditions, day/night: 30/28 °C, 60–80% RH, light: 12 h/12 h). At the cotyledon stage, seedlings were sown in plastic pots (9x9x10cm, 630 ml) filled with classic peat and coconut fibre substrate (Einheitserde®, Sinntal-Altengronau, Germany) and placed in a semi-climate-controlled glasshouse with ambient light (14–11 h light per day), between 25 and 30 °C and 50–70% of relative humidity. Plants were watered every other day.

#### *Insects*

Caterpillars of the velvet armyworm, *Spodoptera latifascia* and the fall army warm, *S. frugiperda*, are commonly found feeding on bean and maize plants respectively, in their native environment and they serve as hosts for the ectoparasitoid species *Euplectrus platyhypenae* [48,49]. Both caterpillar species were initially collected from maize and bean plants in the location in Mexico where wild lima bean seeds were also collected [49,50]. *E. platyhypenae* parasitoids were obtained from parasitized caterpillars of both species in the same location. All insects were transported to the University of Neuchâtel where they were reared in a quarantine facility (26 °C, 60% r.h., 12 h/12 h light/dark). Caterpillars were fed on an artificial diet (Beet Armyworm diet®, Frontier Agri.Science, USA).

Adults of *E. platyhypenae* were kept in 30x30 × 30 cm mesh cages (BioQuip Products, Rancho Dominguez, USA) with drops of honey and humid cotton. For the rearing and oviposition bioassays, single-mated, 2–4 days old females were placed in a Petri dish with one maize leaf humidified by a cotton ball, a drop of honey and a third-instar caterpillar of *S. latifascia* or *S. frugiperda*. Once the caterpillars were parasitized, they were returned to the artificial diet until parasitoid pupation. Upon emergence, adult parasitoids were transferred to mesh cages.

### 2.2 Preparation of rhizobia inoculant

For *P. lunatus*, ∼50 nodules were collected from five *P. lunatus* plants, containing the symbiont *Bradyrhizobium elkanii* (Accession DJB1033-Ballhorn Lab; Portland State University) [16]. Similarly, for *P. vulgaris*, 82 nodules were isolated from four *P. vulgaris* plants in a permaculture garden at the University of Neuchâtel. This local rhizobium strain is yet to be identified. Nodules from each species were collected separately, washed and crushed with a micro pestle in a 2 mL Eppendorf tube. The resulting material was mixed with 1 L of tap water, creating a solution used for inoculating the respective bean species. Bean seeds were sown in 630 ml plastic pots, and upon germination, each plant received a 20 ml dose of the prepared stock solution. Plants were grown under artificial LED light in the university's greenhouse at approximately 25 °C average; 60% relative humidity; and with a photoperiod of L12:D12 and an average light intensity of around 350 μmol *m^−2^ * sec^−1^.

For all subsequent experiments, plant inoculation was carried out by extracting 65 nodules from the rhizobia-reservoir plants, following the same procedure described earlier to obtain the inoculum solution. In the rhizobia treatment (hereafter R+), 20 ml of this solution was added to the soil of pots containing each species, while the non-inoculated group (hereafter, R-) plants received 20 ml of tap water. We supplemented R- *P. lunatus* plants with low dosed fertilizer (10 ml of 0.2% aqueous solution of NPK: 8/8/6%; Maag, Switzerland, Dielsdorf, at one and two weeks after germination) to control for the effects of nitrogen on R+ plants [46].

### 2.3 Experiments

In 2019, we conducted a first series of experiments using wild plants of *P. lunatus*, caterpillars of *S. latifascia* and parasitoid wasps of *E. platyhypenae*. During the COVID-19 pandemic, we lost the colony of *S. latifascia* and depleted our supply of seeds of *P. lunatus*. As it was not possible to travel to Mexico to collect seeds and insects in 2020 a second series of follow-up experiments was conducted using cultivated plants of *P. vulgaris* and caterpillars of *S. frugiperda*. We used the same colony of *E. plathypenae* that was used in the previous experiment.

#### 2.3.1 Evaluation of the impact of rhizobia-bean symbiosis on plant traits

For this experiment, we used 25 lima bean plants (four weeks old), divided into two rhizobia-treatment groups: R+ and R-. Groups of five to six pots with plants of the same treatment were contained within the same tray to avoid cross contamination between treatments. Trays with different treatments were positioned in an alternated layout in the greenhouse. First, we assessed the effect of rhizobia on the concentration of the two main CNGs in lima bean leaves, linamarin and lotaustralin [35]. Five leaflets were collected per each plant, located between the first true leaf and the last fully expanded leaf, in both the R+ and R-treatment groups. Leaves were pooled and flash-frozen in liquid nitrogen and stored at −80 °C for subsequent chemical extraction and analysis (see below). Afterward, we assessed the nodulation success by counting the nodules in the rhizosphere for each plant. Next, we introduced 12-days old *S. latifascia* caterpillars onto ten plants per rhizobia treatment group. T The caterpillars were individually placed in a clip-cage attached to a leaf, with one caterpillar attached to each leaf category (old, middle, young) to distribute damage across the plant. After 24 h, we repeated the leaf collection for the same plants to examine the effects of caterpillar damage. We estimated the aboveground mass by drying the remaining tissues in an oven at 80 °C for 9 days and weighing them.

The emissions of herbivore-induced VOCs were evaluated in six plants per rhizobia treatment to analyze the impact of rhizobia-bean symbiosis on plant volatiles. To collect aboveground VOCs, we placed entire plants inside glass vessels (diameter: 6.5 cm, height: 25 cm). Purified and humidified air was pushed at the base of each vessel at a rate of 0.6 L/min, while emitted volatiles were adsorbed on a Porapak Q® filter connected in a horizontal position at the top of each glass vessel. A vacuum pump was used to extract the air from the system at a rate of 0.5 L/min. Volatiles were collected for 3 h between 10:00 a.m. and 1:00 p.m.

##### Determination of foliar CNGs and emitted plant volatiles

To quantify CNGs, we used a method adapted from two previous studies [51,52]. For each sample (one sample per plant), 20 mg of ground leaf powder were added to 1 ml of ice-cold 70% methanol. The tubes were immediately transferred to a heating plate at 90 °C for 10 min, placed in an ultrasonic bath for 15 min, then centrifuged at 16 000 RCF for 10 min. Finally, 100 μl of the supernatant were diluted in Milli-Q water at a concentration of 1:10 for analysis. The quantification of CNGs was performed using an Acquity UHPLC (ultra-high-performance liquid chromatography) system coupled to a Synapt G2 QTOF mass spectrometer (Waters, Milford, USA) controlled by Masslynx 4.1 (Shlichta et al., 2014).

The volatile compounds emitted by R+ and R-plants previously exposed to herbivory were eluted with 150 μL of dichloromethane and spiked with internal standard (200 ng nonyl acetate) before being analysed by gas chromatography coupled to mass spectrometry (GC-MS; Agilent 7890B–5977B). Specifically, 1.5 μl of each sample were injected in a pulsed splitless mode in the front inlet at 250 °C with helium at a constant flow of 0.9 ml∙min^−1^. Oven temperature was held at 40 °C for 3.5 min and increased to 100 °C at a rate of 5 °C∙min^−1^ followed by a post run hold of 3 min at 250 °C. Compound identification was performed with comparison to NIST library and available authentic standards. Peak areas were standardized by the known concentration of internal standards, and the compound emission was standardized by total aboveground dry biomass. Concentrations were corrected with a response factor of each compound relative to the internal standard. When the response factor was not available for a compound, we used the response factor of the closest compound of the same class, normalized by the molecular mass.

##### Analysis of protein content in leaf tissues

A Bradford assay was used to quantify leaf protein content [53], using a method adapted from Hernandez-Cumplido et al. [44] In a different set of lima bean plants (25 days old), the central leaflet of the first trifoliate leaf of each of 30 plants per treatment was ground in liquid nitrogen and 40 mg of leaf powder was placed in a centrifuge tube to which 400 μL of protein extraction buffer (Tris-HCl 1 M, pH 6.8, 20% sodium dodecyl sulphate (w/v), dithiothreitol 1 M, glycerol 87% and MiliQ water) was added. The samples were vortexed and then incubated for 1 h at room temperature. Subsequently, the samples were incubated in a heating block at ∼100 °C for 2 min, and then centrifugated for 30 min at 16 000 RCF before collecting the supernatant. Finally, 1 μl of the extracted sample was mixed with 799 μL of ddH2O and 200 μL of Bradford solution. The samples were vortexed and then incubated for 5 min before measuring the absorbance at 595 nm. The protein concentration of the samples was determined using BSA standards from 0 to 20 μg.

#### 2.3.2 Assessment of the impact of rhizobia-bean symbiosis on herbivores and parasitoids

Caterpillar performance was measured by individually placing one-day old caterpillars inside clip-cages (BioQuip®, Ø = 25.4 mm; h = 9.5 mm) attached to leaves of four-weeks-old undamaged lima bean plants. The mass of each caterpillar was measured at the start of the experiment and again after five and twelve days of development. To ensure a fresh leaf supply of leaf material for the caterpillars, the clip-cages were moved to different locations on the plant daily. We placed three clip-cages on each plant, covering different leaf stages (old, middle, young). Each experimental setting was replicated 15 times, resulting in a total of 45 caterpillars per rhizobia treatment. Each plant was individually placed in the experimental room at 24 ± 2 °C under lamps (day/night: 12 h/12 h) inside mesh cages. After pupation, we recorded the time from egg hatching to pupation and measured pupal mass.

We conducted a choice experiment to test the preference of *S. latifascia* caterpillars when given the option of R+ and R-leaves. One leaflet from each treatment (obtained from undamaged four-week-old plants) was placed on opposite sides of a Petri dish (90 mm diameter) with a 7-day old caterpillar positioned between them (n = 20 per treatment). After a 24 h period, we measured both the leaf area and damaged area using the application LeafByte® [54].

After 12 days of continuous feeding, we collected 20 caterpillars per rhizobia treatment, weighing between 12 and 36 mg. Each caterpillar was placed individually in a Petri dish containing the following: a mated naive 2–4 days old female *E. platyhypenae*, a drop of honey and a leaflet from the corresponding treatment group of the caterpillar. Female wasps were removed from the dish after four days. We recorded the number of parasitoid eggs, larvae, pupae and adult offspring, development time (time from oviposition to adult emergence), offspring sex ratio and offspring survivorship (offspring adults/number of eggs).

Parasitoid choice for R+ or R- VOCs was tested using a four-arm glass olfactometer with an upward connection for insect-trapping glass bulbs [55]. First, volatile emission was induced by placing three 12-days old *S. latifascia* caterpillars on each plant (25 days old) as described above. For each olfactometer test, the treatments consisted of one R+ plant, one R-plant and two empty glass bottles. Purified and humidified air was injected at the base of each glass vessel at a rate of 0.8 L/min. Wasps were released in groups of 6 individuals, with the help of an insect aspirator, directly in the central chamber of the olfactometer. After 30 min, wasp position in the olfactometer was recorded; wasps that entered a collection bulb or the elbow underneath each bulb were considered as having made a choice, otherwise they were considered as having made “no choice”. Three releases of six wasps were performed before changing the plants and all glass parts. For each repetition, a new set of plants and glass vessels were used, and the position of the plants and the empty vessels were randomly assigned in the olfactometer. Active air extraction was performed with a vacuum pump. The experiment was repeated eight times during two consecutive days between 10:00 a.m. and 6:00 p.m. A second set of experiments was performed with a similar setup but with some modifications. This time, 4-week-old plants were used, as the plants were germinated at the same time and the experiment was performed on week later. The setup was repeated ten times during two consecutive days. Wasps located under the elbow connector to the central chamber were also considered as having made a choice. Between each day of experiment, all glass parts were rinsed with acetone and pentane, then placed in an oven at 250 °C overnight.

#### 2.3.3 Effect of rhizobia-bean symbiosis on extrafloral nectar (EFN) production and its consequences for parasitoids

We transferred 41 four-week-old *P. vulgaris* plants (20 R- and 21 R+) into an experimental room at 24 °C under artificial light (day/night: 12 h/12 h). Fourteen bean plants in each rhizobia treatment were subjected to damage using a metal hole puncher (diameter: 15 mm) to induce the production of EFN (adapted from Heil et al., 2000), while the rest of the plants remained undamaged. Three holes were made in one half of each leaflet, and after 24 h, on the other half. Forty-eight hours after the first damage, EFN from the five youngest leaves was collected with a 5 μl capillary pipette (Vitrex Medical, Herlev, Denmark) from these damaged plants. Additionally, seven (R+) and six (R-) plants were sampled to have information about the differences in EFN induction. Because of the EFN thickness, the microcapillaries were filled with 2 μl of water to allow the EFN to flow within the tube. The volume of EFN retrieved from each plant was measured with a ruler and quantified with a 0–50 °Brix refractometer (ATAGO, Tokyo, Japan).

To assess the direct interaction between plants and parasitoids mediated through EFN, we compared the performance of parasitoids fed with EFN from R+ and R-plants. In a separated set of plants, 0.2 μl of EFN was collected with 1 μl of water from 29 plants induced in the same way for both rhizobia treatments. Diluted nectar was then added in the lid of a Petri dish. We included a similar diet treatment using commercial organic honey (Bio Honig, Germany) instead of nectar as a positive control. In each Petri dish, one naive 2–4 days old female and one naive male parasitoid were allowed to feed on the nectar or honey solution for three days. After that, one third-instar caterpillar of *S. frugiperda* was placed in the Petri dish with a 10 cm piece of maize leaf humidified with a wet cotton ball replaced every two days. After five days, parasitized caterpillars were moved to a clean empty Petri dish containing a piece of maize leaf to continue parasitoid development. Unparasitized caterpillars were discarded. A new third-instar caterpillar was added every five days in the Petri dish containing the parasitoids and this process was repeated until the female parasitoids died. Just before the addition of the next caterpillar, 0.1 μl of extrafloral nectar or honey was collected a second time with 1 μl of water and added in the lid of the Petri dish. Caterpillars in each Petri dish were checked every day and we recorded: the number of laid eggs, the number of larvae, pupae and adults, developmental time (number of days from oviposition to adult emergence), offspring sex ratio and offspring survivorship (offspring adults/number of eggs). The longevity (number of days from adult emergence to death) of each parasitoid parent was recorded under each diet condition, including the presence of a wet cotton ball.

### 2.4 Statistical analyses

For the analyses we used (generalized) linear models in R system, version 4.3.1 (R Core Team 2023) with the package ‘stats’ or ‘lme4’ [56] to explore the effect of rhizobia treatment on plant and insect traits. A summary of the univariate analyses is shown in Table 1. For analysing the composition of the VOC emissions in R+ and R-plants we used a multivariate redundancy analysis (package ‘vegan’). The effects of explanatory variables of all models were obtained with an ANOVA type II and posthoc analyses were performed with a Tukey method using package ‘emmeans’. Whenever a GLM(M) was overdispersed (dispersion parameter >2), an observation level random factor was added to control for overdispersion.

**Table 1:** Summary of the univariate analyses used for exploring differences in plant and insect traits between rhizobia-inoculated and not inoculated bean plants. We used linear models with normal error distribution (LM), generalized linear models (GLM) and mixed models (LMM, GLMM). The error distribution family used for GLMs or GLMMs is indicated in parentheses, for the VOCs analyses we adjusted p values for the multiple testing using a false discovery rate correction.

Response variable	Explanatory variable	Model	Random factor
Shoot biomass	Rhizobia treatment + nodule number	LM	
Leaf protein	Rhizobia treatment + nodule number	LM	
CNGs	Rhizobia treatment + herbivory treatment + nodule number	LMM	Plant ID
VOCs	Rhizobia treatment	GLM (Gamma, log link)	
Caterpillar mass gain	Rhizobia treatment + Initial mass	LM	
Pupal mass	Rhizobia treatment	LM	
Caterpillar development	Rhizobia treatment	Cox model	
Leaf damage	Rhizobia treatment	LMM	Petri dish ID
Parasitoid eggs, adults	Rhizobia treatment	GLM (Poisson)	
Parasitoid sex ratio	Rhizobia treatment	GLM (Binomial)	
Offspring survivorship	Rhizobia treatment	GLM (Binomial)	
Parasitoid olfactometer	Arm treatment + block	GLMM (Poisson)	Replicate ID
EFN volume	Rhizobia * damage treatment	GLM (Gamma, log link)	
EFN Brix	Rhizobia * damage treatment + EFNvolume	GLM (Gamma, log link)	
Parasitoid survival	Diet treatment	Cox model	

## 3 Results

1. Evaluation of the impact of rhizobia-bean symbiosis on plant traits.

Roots from control, R-, plants did not develop any nodules, meanwhile roots of rhizobia-inoculated R+ plants had formed between 210 and 780 nodules per plant. The rhizobia inoculation treatment did not influence aboveground plant biomass (F_(1,18)_ = 0.18, P = 0.68). However, the number of nodules in the root system of R+ plants was positively associated with aboveground biomass (F_(1,10)_ = 20.17, P = 0.001). No significant difference was found for leaf soluble protein content between R+ and R-plants. R+ plants had on average 79.6 ± 9.1 mg of protein per gram of dry mass (DM) compared to 99.3 ± 5.3 mg/g DM for R-plants (F_(1,28)_ = 3.50, P = 0.072), and the soluble protein content in leaves was not correlated with the number of nodules in R+ plants (F_(1,13)_ = 1.90, P = 0.191).

**Fig. 1:**
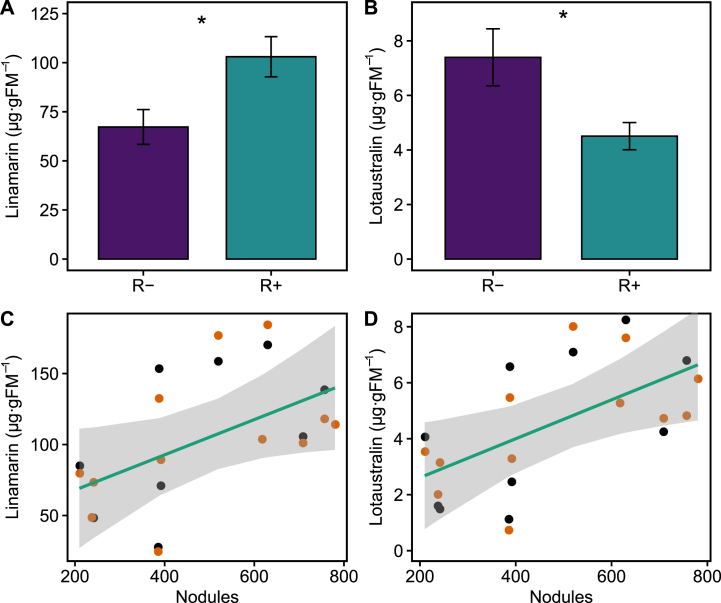
**Effect of rhizobia on CNGs.** Shown are the mean concentrations (μg per gram of fresh leaf mass) of linarmarin (A) and lotaustralin (B) in in plants inoculated with rhizobia (R+; green bars) or not inoculated (R-; purple bars). Asterisks indicate level of significance: *p < 0.05, **p < 0.01 and ***p < 0.001. Scatterplots in panels (C) and (D) show correlations between the concentration of linamarin and lotaustralin and of the number of nodules, respectively. Dot colours indicate damage treatment (black: before damage, orange: after damage). *N* = 36. The regression line (95% C.I.) shows the estimated coefficient from a mixed model that considers the effects of rhizobia treatment, herbivory treatment and the repeated measures design (see Table 1). (For interpretation of the references to colour in this figure legend, the reader is referred to the Web version of this article.)

**Fig. 2:**
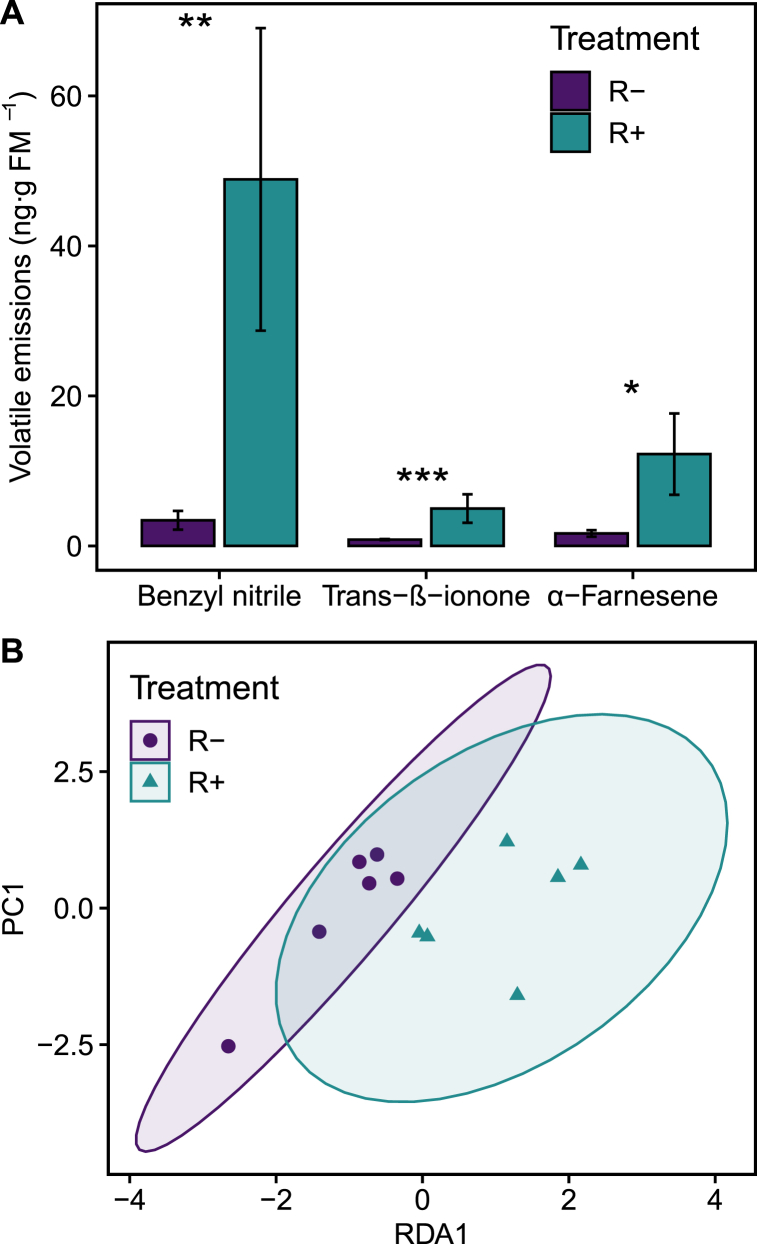
**Effects of rhizobia on VOCs emissions of lima bean plants.** A) Barplot showing means (±1 SEM) of emissions of three compounds that are present in significantly different concentrations across plants inoculated with rhizobia (R+; green bars) or not (R-; purple bars)). *N* = 12. Asterisks indicate level of significance: *p < 0.05, **p < 0.01 and ***p < 0.001. B) RDA analysis on the composition of emitted volatiles between plants inoculated with rhizobia (R+; green ellipse and triangles) or not (R-; purple ellipse and dots). Ellipses represent 95% confidence intervals around the cloud of points. (For interpretation of the references to colour in this figure legend, the reader is referred to the Web version of this article.)

Concentrations of plant defensive cyanogenic glycosides (CNGs) linamarin and lotaustralin were significantly different between the two rhizobia treatments (Fig. 1). Across all plants, linamarin concentration was higher in R+ plants than in R-plants (χ^2^_(1)_ = 3.84, P = 0.050; Fig. 1A), but herbivory treatment did not affect linamarin concentrations (χ^2^_(1)_ = 1.14, P = 0.29). On the contrary, lotaustralin concentration was almost 40% lower in R+ plants than in R-plants (χ^2^_(1)_ = 3.84, P = 0.050; Fig. 1B), and again, it did not change after herbivory (χ^2^_(1)_ = 0.008, P = 0.92). Yet, the concentration of both linamarin and lotaustralin was positively correlated with the number of root nodules (χ^2^_(1)_ = 4.41, P = 0.036, Fig. 2C; χ^2^_(1)_ = 6.68, P = 0.0098, Fig. 2D; respectively).

Total VOCs emission remained similar across R- and R+ plants (χ^2^_(1)_ = 0.041, P = 0.84). However, compared against R-plants, R+ plants emitted significantly higher amounts of some compounds (Fig. 2A; Table S1) such as, benzyl nitrile (χ^2^_(1)_ = 13.14, P = 0.009), α-farnesene (χ^2^_(1)_ = 10.66, P = 0.035) and *trans*-β-ionone (χ^2^_(1)_ = 20.46, P = 0.0002). Accordingly, the multivariate redundancy analysis showed that the composition of the emitted volatile blend was different between both treatments (χ^2^_(1)_ = 18.52, P > 0.0001; Fig. 2B).

2. Assessment of the effect of rhizobia-bean symbiosis on herbivores and parasitoids.

**Fig. 3:**
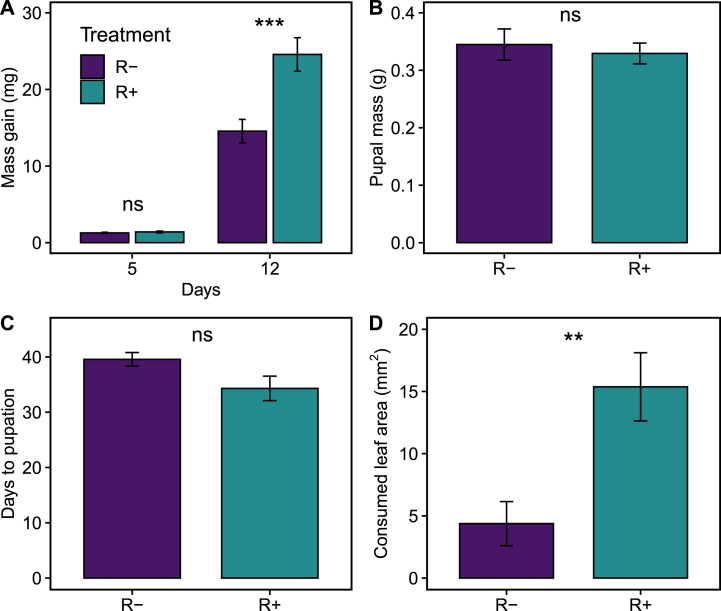
**Effects of rhizobia on the performance of *Spodoptera latifascia* caterpillars.** Barplots in all panels show means (±1 SEM) of larval mass gain of caterpillars at five and twelve days after experiment started (*N* (day 0–5) = 90, *N* (day 12) = 32) (A), *S. latifascia* pupal mass (N = ) (B), developmental time from egg hatching to pupation of *S. latifascia* (N = ) (C),and consumed leaf area by *S. latifascia* caterpillars after 24 h of feeding (N = ) (D), on leaves for non-inoculated (R-; purple bars) and inoculated plants (R+; green bars). Asterisks indicate level of significance: *p < 0.05, **p < 0.01 and ***p < 0.001. (For interpretation of the references to colour in this figure legend, the reader is referred to the Web version of this article.)

Lima bean plants that were inoculated influenced the interaction with *S. latifascia* caterpillars and *E. platyhypenae* parasitoids. Caterpillars gained similar amount of mass after five days of feeding on R- or R+ bean plants (χ^2^_(1)_ = 0.74, P = 0.39; Fig. 3A), but after 12 days caterpillars fed on R+ plants were 60% heavier than those fed on R-plants (χ^2^_(1)_ = 11.75, P < 0.001; Fig. 3A). Pupal mass was not significantly different between the two treatments (χ^2^_(1)_ = 0.24, P = 0.62; Fig. 3B). Yet, caterpillars from the R+ treatment developed faster than R-caterpillars, pupating on average 7.24 days earlier than R-caterpillars (χ^2^_(1)_ = 8.4, P = 0.004; Fig. 3C). In the choice experiment, caterpillars ate more than three times the amount of tissue from R+ leaves than from R-leaves (χ^2^_(1)_ = 9.87, P = 0.002; Fig. 3D) with no influence of leaflet size (χ^2^_(1)_ = 0.53, P = 0.47).

**Fig. 4:**
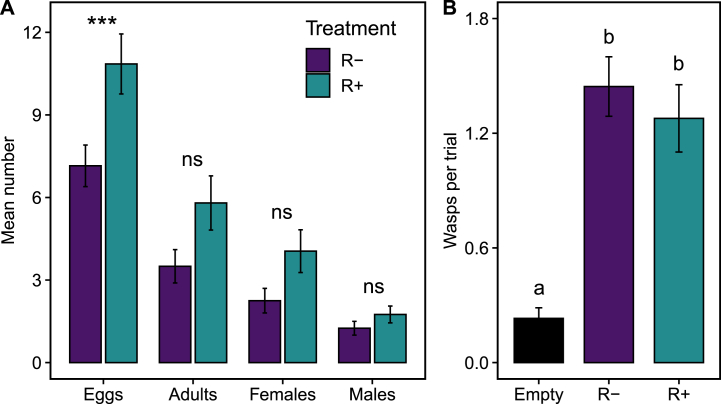
**Effects of rhizobia inoculation on *Euplectrus platyhypenae* parasitoids.** A) Mean number of eggs and adults, females and males of *E. platyhypenae* that were laid on or emerged from *S. latifascia* caterpillars fed on lima bean plants inoculated (R+) or not (R-) with rhizobia. *N* = 40. B) Mean choice responses by *E. platyhypenae* females to the different odour sources offered in a four arms olfactometer. The bar plots show only the wasp that made a choice and values for both control arms (empty vessels) were averaged in each trial. Error bars (±1 SEM). Asterisks indicate level of significance: *p < 0.05, **p < 0.01 and ***p < 0.001; and different letters indicate comparisons with p < 0.05.

Parasitoids traits improved when they were reared on caterpillars from inoculated plants (Fig. 4). The number of parasitoid eggs laid per caterpillar was 57% higher on caterpillars fed with R+ plants than in R-plants (χ^2^_(1)_ = 15.32, P < 0.001; Fig. 4A). There was a positive correlation between caterpillar mass and the number of parasitoid eggs laid on them (χ^2^_(1)_ = 9.39, P = 0.002). However, when we simultaneously tested the effects of rhizobia treatment and caterpillar mass on the number of laid eggs, only the rhizobia treatment was relevant (χ^2^_(1)_ = 7.65, P = 0.006; χ^2^_(1)_ = 1.73, P = 0.19, respectively). The number of all emerged adults and females was slightly higher on caterpillars fed on R+ plants than those fed on R-plants, but the differences were marginally non-significant (adults: χ^2^_(1)_ = 3.13, P = 0.077; females: χ^2^_(1)_ = 3.68, P = 0.055; Fig. 4A). The number of emerged males was not significantly different between both treatments (χ^2^_(1)_ = 1.67, P = 0.20; Fig. 4A). Overall the sex ratio of emerging offspring adults was female biased and not significantly different between R+ and R-treatments (global mean: 66% ± 4% of females; χ^2^_(1)_ = 0.61, P = 0.43). The survivorship of offspring from egg to adult was not statistically different between treatments (global mean: 56.5% ± 5.8%; χ^2^_(1)_ = 0.70, P = 0.40).

Results from the olfactometer experiments showed an attraction to bean plants and an effect of the experimental block on the number of wasps choosing any arm (χ^2^_(2)_ = 66.65, P < 0.0001; χ^2^_(1)_ = 9.43, P = 0.002, respectively). In the first experimental block, only 38.9% percent of the mated-female wasps made a choice among the four arms. The number of choosing wasps increased to 64.8% in the second experimental block. Overall, wasps preferred arms with plants over arms with empty vessels, but no distinction was made between rhizobia treatment (Empty vs. R-: P < 0.0001, Empty vs. R+: P < 0.0001, R-vs. R+: P = 0.74; Fig. 4B), independently of the blocking effect (Treatment by Block interaction: χ^2^_(1)_ = 0.98, P = 0.61).

3. Effect of rhizobia on EFN production and its consequences for parasitoids.

**Fig. 5:**
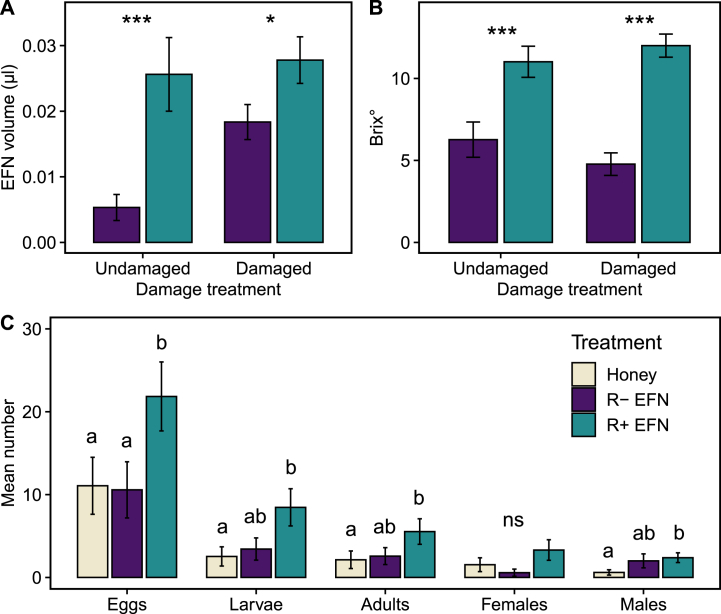
**Effects of rhizobia inoculation of common bean plants (*Phaseolus vulgaris*) on extrafloral nectar (EFN) and parasitoids (*Euplectrus platyhypenae*) fed with different carbohydrate sources.** EFN volume (A) and brix° as an index of carbohydrate content (B) from *P. vulgaris* plants inoculated with rhizobia (R+) or not (R-) with or without mechanical damage. Mean values for panel B correspond to the estimated marginal means from the linear model. C) Mean number of eggs, larvae, and adults, female and male offspring of *E. platyhypenae* fed with commercial honey, extrafloral nectar from bean plants inoculated (R + EFN) or not (R- EFN) with rhizobia. *Spodoptera frugiperda* caterpillars were used as host with a leaf maize diet. N = 35. Asterisks indicate level of significance: *p < 0.05, **p < 0.01 and ***p < 0.001; and different letters indicate comparisons with p < 0.05.

Overall, rhizobia inoculation and the damage treatment modified the volume of EFN produced by the plants (Fig. 5; χ^2^_(1)_ = 17.83, P < 0.0001; χ^2^_(1)_ = 7.67, P = 0.01; respectively), with damaged and undamaged R+ plants producing 381% and 51% more nectar than R-plants, respectively (see significant interaction between the rhizobia and damage treatments, χ^2^_(1)_ = 6.63, P = 0.01; Fig. 5A). Overall, R+ plants produced 140% more Brix counts than in R-plants (χ^2^_(1)_ = 70.3, P < 0.0001; Fig. 5B), and EFN content positively correlated with the volume of EFN (χ^2^_(1)_ = 16.77, P < 0.0001). The effects of damage treatment and its interaction with rhizobia treatment on the EFN Brix degrees were not statistically significant (χ^2^_(1)_ = 0.69, P = 0.41; χ^2^_(1)_ = 0.89, P = 0.35; respectively).

The nectar produced by *P. vulgaris* plants with or without the rhizobia symbiont affected the parasitoid species *Euplectrus platyhypenae*. The source of diet for adult parasitoids (commercial honey, extrafloral nectar (EFN) from R-plants or R+ plants) influenced wasp oviposition on *S. frugiperda* caterpillars (χ^2^_(2)_ = 62.8, P < 0.0001; Fig. 5C). Parasitoids fed with R + EFN laid more eggs than parasitoids fed with R- EFN or honey (P < 0.0001 for both comparisons). The number of parasitoid larvae and emerging adults was the highest for the R + EFN treatment (χ^2^_(2)_ = 7.57, P = 0.023; χ^2^_(2)_ = 6.29, P = 0.043; respectively, Fig. 5C), but only the difference between R + EFN and honey diets was statistically significant (larvae: P = 0.017, adults: P = 0.033). The number of emerging females was not significantly different between the diet treatments (χ^2^_(2)_ = 4.62, P = 0.099), but there were differences in the number of male offspring (χ^2^_(2)_ = 8.12, P = 0.017). More males emerged from the R + EFN than from the honey diet (P = 0.015). Adult sex ratio shown differences among diet treatments (χ^2^_(2)_ = 12.11, P = 0.0023). The sex ratio was more female biased in honey (61% females) than in R- EFN diet (21% females; P = 0.004) and in R + EFN (49% females) than in R-EFN (P = 0.027); but there were no significant differences between R + EFN and honey diets (P = 0.38). The survival time of both mothers and fathers was not significantly affected by their diet (χ^2^_(2)_ = 1.73, P = 0.42; χ^2^_(2)_ = 1.91, P = 0.39; respectively) averaging 19 ± 1.63 and 19.47 ± 2.14 days for females and males, respectively. The survivorship of offspring from egg to adult emergence was not statistically different among the diet treatments of the parents (global mean: 25.9% ± 5.5%; χ^2^_(2)_ = 2.27, P = 0.32).

## 4 Discussion

Nitrogen fixing rhizobia modify plant metabolism in multiple ways, in turn impacting higher trophic level organisms as it has been previously observed and predicted [12,17,46,57]. We particularly show that inoculated lima bean plants favoured *S. latifascia* caterpillars mass gain, and development time, most likely through increased nutritional quality [58]. Secondly, we observed diverse positive effects on the parasitoids, as a result of the indirect interactions with the plants, mediated by the host caterpillar, and direct interactions through the consumption of extrafloral nectar. Below, we discuss the context-dependency of these rhizobia-mediated direct and indirect effects.

Regarding the impacts of rhizobia symbiosis on herbivores, it is hypothesized that while plants benefit from additional nitrogen for enhanced growth and immunity, they may also exhibit heightened defence mechanisms when in symbiosis [15,17]. The effect could be directly influenced by the additional benefits conferred by the symbiosis. Indeed, while allocating resources to defence is assumed to be generally costly for plants [59], leading to trade-offs between growth and defence, beneficial soil microbes have been shown to alleviate, or even remove, these trade-offs [60,61]. This is particularly relevant when considering the resource abundance in local environments, as it likely to determine the extent of the trade-off between defence and growth [62]. Rhizobia-mediated higher CNG levels in lima bean plants could have counteracted the benefits of a more nutritive food source. Indeed, Kempel et al. [14] observed that *Spodoptera littoralis* performed significantly better on rhizobia inoculated plants than on non-inoculated plants, but only when the herbivore performance was tested on the acyanogenic strains of white clover (*Trifolium repens*). In our study, we found that some performance variables of *S. latifascia* were positively impacted by rhizobia inoculation, notably time to pupation and weight gain after 12 days. Here, effects of the rhizobia on the cyanogenic glycosides were mixed, with linamarin increasing and the lotaustralin decreasing with microbes; although the global amount of CNGs was higher in R+ plants. Moreover, we observed no effect of the microbes on soluble proteins, contrary to previous findings [17,63]. This implies that the fertilizer applied to R-plants might have compensated for the absence of rhizobia. However, Thamer et al. [15] did not find differences in soluble protein levels in leaves between inoculated and non-inoculated lima bean plants when both treatments were supplemented with a low amount of fertilizer.

Furthermore, we observed that *S. latifascia* caterpillars preferred rhizobia-inoculated plants. Leaves from plants with rhizobia were darker than leaves from R-plants, which could indicate a herbivore preference for colour rather than for nutritional quality. However, in choice tests with the Mexican bean beetle (*Epilachna varivestis*), Thamer et al. [15] found no differences in leaf consumption between rhizobia-colonized plants and not colonized under light or dark conditions, indicating that leaf nutritional quality and concentration of defensive compounds is likely more important than variation in colour [15,64]. Hence, either the effects of the rhizobia on leaf nutritional quality outweighed the effect of higher doses of linamarin, or *S. latifascia* caterpillars are generally insensitive to CNGs as previously shown for the same species [35], or for *Spodoptera littoralis.* [65,66] Both hypotheses are non-mutually exclusive and future research on his system could also use acyanogenic lines and measure other variables, such as amino acid content, or carbon-to-nitrogen ratio [16], to disentangle them.

To date, research on the effect of mutualistic bacteria on higher trophic levels has mainly focused on herbivores (but see Refs. [17,17,57,[67], [68], [69]]). In our study, we aimed to expand upon this knowledge by exploring how the symbiosis between *Phaseolus* species and rhizobia influences the interaction between herbivores and their parasitoid. We found that the rhizobia-bean interaction affected parasitoids in two ways: indirectly through host caterpillar-mediated effects, or directly, through changes in the quality and quantity of the extrafloral nectar produced bean plants. Interestingly both trophic pathways showed positive effects on parasitoids. Parasitoids egg and offspring numbers were higher when the host caterpillars were fed with rhizobia-inoculated *P. lunatus* plants. Egg oviposition was also positively correlated with caterpillar body mass, but the positive effect of rhizobia inoculation was more relevant. Considering that *E. platyhypenae* females inject venom to halt development in their host and can feed on caterpillar hemolymph [70], they have the opportunity to evaluate host quality before ovipositing. This suggests that the parasitoid preference for caterpillars grown in R+ plants arises from a more suitable caterpillar physiology rather than merely its size. Yet, developing parasitoids have been shown to be affected by the toxins ingested by their host [21,71]. For instance, Chabaane et al. [72] found that *S. latifascia* caterpillars sequestered capsaicin from chili plants, which negatively affected the development of *Euplectrus platyhypenae* parasitoids. Information on the ability of Lepidopteran species to sequester CNGs is mixed. For example, caterpillars of *Zygaena* butterflies were found to accumulate 30% of the ingested CNGs in their hemolymph and can even synthesise their own CNGs [65]. In contrast, *S. littoralis* caterpillar fed with leaves of *Lotus japonicus* excreted most of the ingested CNGs (linamarin and lotaustralin), with no evidence of sequestration [66]. We thus speculate that *S. latifascia* caterpillars can also excrete untransformed CNGs, while being tolerant to these compounds. This could explain why parasitoids also perform better on caterpillars fed on rhizobia plants, likely constituting a more nutritious and not toxic host.

Going further, we also show that the bean-rhizobia symbiosis not only affects parasitoids indirectly via the host caterpillar, but also directly by providing a high-quality food source. Indeed, we found that rhizobia-inoculated *P. vulgaris* plants produced more and richer extrafloral nectar than non-inoculated plants. In turn, when *E. plathypenae* parasitoids fed on nectar from rhizobia-inoculated bean plants laid more eggs which resulted in more offspring than on rhizobia-free plants, or when they were fed with commercial honey. Floral and extrafloral nectar is known to provide a food source rich in carbohydrates that complements the diet of natural enemies of herbivores including, predators and parasitoids [24,26,73]. In agricultural systems, floral nectar is often restricted due to the confinement of flowers to field margins and semi-natural zones [74]. As a result, integrating intercropping with rhizobia-carrying legumes that release elevated levels of extrafloral nectar could bear significant importance in this context, serving to enhance the population of natural enemies.

Along those lines, we also hypothesized that rhizobia could influence parasitoids behaviour via modifications of the blend of volatile organic compounds (VOCs), which are well known to play a role in the recruitment of predators and parasitoids by plants [75]. Yet, we found that rhizobia did not change the total amount of VOCs, as was previously shown in *P. lunatus* [16], and concurrently, wasps could not discriminate by the two rhizobia treatments. That said, the rhizobia treatment modified the finely grained structure of the VOCs blend. For instance, the nitrogenated VOC benzyl nitrile was ten times more abundant in R+ than in R-plants, suggesting that the symbiosis with rhizobia, allows bean plants to access more nitrogen and synthetize and release larger amounts of nitrogen-based compounds. Similarly, other VOCs, such as α-farnesene and *trans*-β-ionone were more abundant in rhizobia plants. Yet, these differences were likely not strong enough to change *E. platyhypenae* behavioural responses. We thus conclude that, while Hay-Roe et al. [48] suggested that certain individual VOCs, particularly those capable of releasing hydrogen cyanide, such as benzyl nitrile emitted by stargrass, could modify the behaviour of *E. platyhypenae*, the experimental conditions in which the parasitoids were tested here did not generate enough variance to spur and detect behavioural changes.

In conclusion, we found that the symbiotic relationship between rhizobia and bean plants has profound effects on multiple trophic levels. We suggest that the effects of rhizobia on herbivores and parasitoids are likely mediated by leaf nutritional quality and, to a lesser extent, by chemical defences, since the caterpillar species appears well adapted to consumption of CNGs. These findings emphasize the complexity of multitrophic interactions and highlight the potential implications for ecological interactions and biological control strategies. Future studies should examine how the degree of herbivore specialization and chemical counter-adaptations (sequestration, detoxification, excretion) determine the outcome of the rhizobia-plant-herbivore interaction and its consequences for the third trophic level.

## Data availabitlty statement

The data and code associated to this article are published in the zenodo repository at https://doi.org/10.5281/zenodo.10229336.

## CRediT authorship contribution statement

**Carlos Bustos-Segura:** Writing – review & editing, Writing – original draft, Validation, Methodology, Investigation, Formal analysis, Conceptualization. **Adrienne L. Godschalx:** Writing – review & editing, Methodology, Investigation, Formal analysis, Conceptualization. **Lucas Malacari:** Writing – review & editing, Methodology, Investigation, Formal analysis. **Fanny Deiss:** Writing – review & editing, Methodology, Investigation, Formal analysis. **Sergio Rasmann:** Writing – review & editing, Resources. **Daniel J. Ballhorn:** Writing – review & editing, Resources. **Betty Benrey:** Writing – review & editing, Writing – original draft, Supervision, Project administration, Methodology, Funding acquisition, Conceptualization.

## Declaration of generative AI and AI-assisted technologies in the writing process

During the preparation of this work the authors used ChatGpt in order to improve language. After using this tool/service, the authors reviewed and edited the content as needed and takefull responsibility for the content of the publication.

## Declaration of competing interest

The authors declare that they have no known competing financial interests or personal relationships that could have appeared to influence the work reported in this paper.
